# Contact and Fumigant Activities of *Citrus aurantium* Essential Oil against the Stable Fly *Stomoxys calcitrans* (Diptera: Muscidae)

**DOI:** 10.3390/plants11091122

**Published:** 2022-04-21

**Authors:** Tanasak Changbunjong, Sookruetai Boonmasawai, Sivapong Sungpradit, Thekhawet Weluwanarak, Arpron Leesombun

**Affiliations:** 1 Department of Pre-Clinic and Applied Animal Science, Faculty of Veterinary Science, Mahidol University, Nakhon Pathom 73170, Thailand; tanasak.cha@mahidol.edu (T.C.); sookruetai.boo@mahidol.edu (S.B.); sivapong.sun@mahidol.edu (S.S.); 2 The Monitoring and Surveillance Center for Zoonotic Diseases in Wildlife and Exotic Animals (MoZWE), Faculty of Veterinary Science, Mahidol University, Nakhon Pathom 73170, Thailand; thehawet.wel@mahidol.edu

**Keywords:** *Citrus aurantium*, contact toxicity, fumigant toxicity, insecticide, stable flies, vector

## Abstract

The stable fly, *Stomoxys calcitrans* (L.), is a cosmopolitan hematophagous fly of medical and veterinary importance. It is widely considered a major livestock pest that can cause significant economic losses. This study aimed to evaluate the insecticidal activity of *Citrus aurantium* (L.) essential oil against *S. calcitrans* based on contact and fumigant toxicity tests. Chemical analysis by gas chromatography-mass spectrometry of the essential oil showed the dominance (93.79%) of limonene in the total essential oil composition. Furthermore, the insecticidal test results showed that the mortality of flies increased with concentration and time within 24 h of exposure. In the contact toxicity test, the median lethal dose was 105.88 µg/fly, while the 90% lethal dose was 499.25 µg/fly. As for the fumigant toxicity test, the median lethal concentration was 13.06 mg/L air, and the 90% lethal concentration was 43.13 mg/L air. These results indicate that *C. aurantium* essential oil exhibits insecticidal activity against *S. calcitrans*. Therefore, it can be used as an alternative to synthetic insecticides for achieving stable fly control.

## 1. Introduction

The stable fly, *Stomoxys calcitrans* (Linnaeus, 1758) (Diptera: Muscidae), is a cosmopolitan hematophagous fly of medical and veterinary importance. This species is considered a harmful pest affecting pets, livestock, wildlife, and even humans. Stable flies have a direct effect on animal health and can lead to economic losses, especially in livestock in which they can cause disturbance, skin lesions, blood loss, decreased food intake, reduced weight gain, and decreased milk production [1]. Stable flies act as mechanical vectors of various pathogens, including bacteria, helminths, protozoa, and viruses [1]. In the US, *S. calcitrans* is considered an economic pest of cattle industries, capable of producing annual losses greater than USD 2 billion per year [2]. In Thailand, *S. calcitrans* is reported to be a vector of pathogens, namely, *Trypanosoma evansi*, which causes trypanosomosis or surra in horses, cattle, and buffaloes, and *Anaplasma marginale*, which causes anaplasmosis in cattle and buffaloes [3,4]. It has recently been reported that this species is related to epidemic outbreaks of lumpy skin disease virus, causing lumpy skin disease in cattle within the country [5]. 

Although there are several management options for controlling stable flies, such as insecticides, biological control, sterile insect techniques, physical control, cultural control, and integrated pest management, at present stable fly control still relies on the use of conventional insecticides. Synthetic pyrethroid insecticides have been proven to be effective against these flies [6]. However, the continuous or repeated applications of these insecticides often lead to resistance in insect populations. For instance, the resistance of stable flies to pyrethroids has been reported in many countries, such as Brazil [7], France [8], Germany [9], the United States [10], and Thailand [8]. For this reason, an alternative to conventional synthetic insecticides using plant-derived products has become a popular research topic in recent years [11,12,13]. The use of plant essential oils, which are secondary metabolites produced by plants, is an effective method in pest management programs [14,15]. Many groups of plant secondary metabolites, including alkaloids, phenols, saponins, and terpenes, are widely considered to be the most promising compounds for insect pest control [15]. Several plants contain essential oils that have been reported to show a wide range of biological activities against insect pests. For example, rosemary (*Rosmarinus officinalis*) in the family Lamiaceae showed contact and fumigant toxicity as well as repellency effects against brown-banded cockroach (*Supella longipalpa*) [16], while Kaffir lime (*Citrus hystrix*) in the family Rutaceae had contact toxicity effects against blow flies (*Chrysomya megacephala*, *C. rufifacies*, and *Lucilia cuprina*) and house fly (*Musca domestica*) [17].

*Citrus aurantium* (Linnaeus, 1753), also known as bitter orange, sour orange, Seville orange, or bigarade, is a plant belonging to the family Rutaceae and is native to Southeast Asia [18,19]. This plant has numerous pharmacological properties, including anticancer, antianxiety, anti-obesity, antibacterial, antioxidant, insecticidal, and antidiabetic activities [19,20]. The essential oil from this plant, which shows insecticidal activity, contains limonene (terpenes group) as its main component [20,21,22,23]. Moreover, the essential oil from *C. aurantium* has been found to have insecticidal activity against various insect pests, including larvae of cotton leafworm (*Spodoptera littoralis*) [24], larvae of tomato leafminer (*Tuta absoluta*) [24,25], larvae of mosquito (*Anopheles stephensi*) [22], rusty grain beetle, (*Cryptolestes ferrugineus*), red flour beetle (*Tribolium castaneum*), book louse (*Liposcelis bostrychophila*) [23], house fly (*M. domestica*) [21], cowpea seed beetle (*Callosobruchus maculatus*) [26], and silverleaf whitefly (*Bemisia tabaci*) [27]. However, the insecticidal activity of the *C. aurantium* essential oil against stable flies has not yet been reported. Therefore, the aim of this study was to evaluate the insecticidal activity of the essential oil from *C. aurantium* against *S. calcitrans* by contact and fumigant toxicity tests.

## 2. Results

### 2.1. Essential Oil Extraction and Analysis

**Figure 1:**
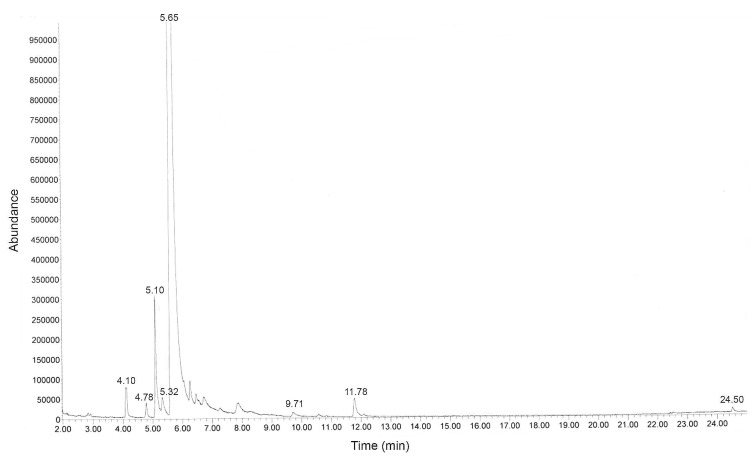
Gas chromatography-mass spectrometry (GC-MS) chromatogram of *Citrus aurantium* essential oil.

The yield of essential oil obtained from fresh peels of *C. aurantium* was 3.31% (*v*/*w*). The oil was clear, colorless and had a pH of 5, a density of 0.84 g/mL at 20 °C, and a refractive index of 1.47. The chemical composition of *C. aurantium* essential oil was determined by GC-MS, and a total of eight compounds were identified, representing 97.79% of the total composition (Figure 1, Table 1 and Figure S1). The main compound was limonene (93.79%) with a concentration of 3.3 mg/mL.

### 2.2. Contact Toxicity Test 

The contact activity of the *C. aurantium* essential oil against *S. calcitrans* was observed among the different concentrations of essential oil at 24 h after treatment. Negative control (acetone) was used to validate the test if there was no insecticidal activity against the flies, while the positive control (cypermethrin 1%) was used to validate the test as an insecticidal agent. The essential oil treatments at 42, 84, and 210 µg/µL and acetone presented low or no insecticidal activity compared to the essential oils with concentrations of 420 and 840 µg/µL and cypermethrin. The essential oil at a concentration of 420 µg/µL showed insecticidal activity similar to cypermethrin at 24 h after treatment, whereas the oil at 840 µg/µL concentration showed insecticidal activity similar to cypermethrin from 1–24 h after treatment (Table 2). The interaction between the concentration and time was statistically significant on *S. calcitrans* mortality (time, F(_2.51, 35.10_) = 21.82, *p* < 0.001; treatment, F(_6, 35.10_) = 112.71, *p* < 0.001; treatment × time, F(_15.04, 35.01_) = 3.58, *p* < 0.001). The toxicity values of *C. aurantium* against *S. calcitrans* were evaluated by LD_50_ and LD_90_ at 24 h after treatment, and the resulting values were 105.88 and 499.25 µg/fly, respectively (Table 3).

### 2.3. Fumigant Toxicity Test

The fumigant activity of the *C. aurantium* essential oil against S. *calcitrans* was observed among the different concentrations at 24 h after treatment. The negative control (acetone) was used to validate the test when there was no insecticidal activity against the flies, while the positive control (cypermethrin 1%) was used to validate the test as insecticidal agent. The negative control and treatments of the essential oils at 0.84, 4.20, 8.40, and 16.80 mg/L air presented low or no insecticidal activity compared to that at 25.20 mg/L air and cypermethrin. The essential oil at 25.20 mg/L air showed insecticidal activity similar to cypermethrin from 2 to 24 h after treatment (Table 4). The interaction between the concentration and time was statistically significant for S. *calcitrans* mortality (time, F_(1.90, 26.65)_ = 13.20, *p* < 0.001; treatment, F_(6, 26.65)_ = 74.73, *p* < 0.001; treatment × time, F_(11.42, 26.65)_ = 2.35, *p* < 0.05). The toxicity values of *C. aurantium* against *S. calcitrans* were evaluated by LC_50_ and LC_90_ at 24 h after treatment, and the resulting values were 13.06 and 43.13 mg/L air, respectively (Table 5). 

## 3. Discussion

In this study, we reported for the first time the insecticidal activity of essential oil extracted from *C. aurantium* peel against the stable fly, *S. calcitrans*. Although the specimens used in this study were directly collected from wild populations and not from laboratory colonies, our results could be used as a baseline for further studies regarding the development of natural products for stable fly control. The advantages of using field specimens include convenience and the generation of test results from the target population [28]. On the other hand, the disadvantage of using field specimens is that the insecticidal activity test results may fluctuate due to factors related to the age and/or physiological status of the insects [28]. For instance, several studies used wild-caught stable flies for testing insecticide susceptibility or resistance [7,13,29].

The monoterpenes are the main secondary metabolites found in *C. aurantium* essential oil [30]. They process several functions in plant physiology and cell membranes and are attributed to biological and medical benefits, including antioxidant, antibacterial, anticancer, antidiabetic, anti-obesity, and anxiolytic effects. They also play a role in the defense of plants against microorganisms and insects [19,20,31,32]. The present study demonstrated that eight compounds represented 97.79% of the total composition of *C. aurantium* essential oil by GC-MS. Limonene was the major constituent and representative of 93.79% of the total oil and concentration of the 3.3 mg/mL sample. Our findings are in accordance with previous studies indicating that *C. aurantium* essential oil extracted from peel contains amounts of limonene ranging from 49–94% [19,20,22,30]. Limonene is the major component in *Citrus* spp. The essential oil of *Citrus reticulata* consists mainly of limonene (85.10%), sabinene (2.49%), linalyl acetate (2.00%), and copaene (1.80%) [33]. *Citrus lemon* essential oil contains limonene (43.07%) followed by β-pinene (12.61%), gamma terpinene (11.48%), α-terpineol (7.20%), α-pinene (3.39%), myrcene (1.87%), geraniol (1.48%), and α-terpinene (1.32%) [34], whereas *Citrus sinensis* essential oil contains limonene (73.24%), α-pinene (5.86%), and myrcene (4.45%) [35]. The differences in chemical composition and amounts of constituents in *C. aurantium* essential oil may be due to the ecological zone, climate, time of harvesting, genetic results, vegetative stage, and extraction processes [19,36,37].

We determined the mortality of stable flies through contact and fumigation toxicity tests. The contact toxicity test is a method to kill a target species upon direct contact, while the fumigant toxicity test is a method performed on a target species in a gaseous phase [38]. In this study, the essential oil at low concentrations showed mortality similar to that of the negative control, whereas the essential oil at high concentrations showed mortality similar to that of the positive control (cypermethrin 1%), in both contact and fumigant toxicity tests. In addition, when the exposure time after treatment increased, the percentage of mortality also increased. For instance, the essential oil concentration at 420 µg/fly showed significant contact toxicity against stable fly at 24 h after treatment, whereas the concentration at 25.2 mg/L air showed significant fumigant toxicity against stable fly when the exposure time increased to the second hour after treatment. The results indicated that the efficacy of the essential oil improved with increasing doses and exposure times; therefore, insect mortality was influenced by concentrations and times. Many plant-derived essential oils, such as the essential oils from tea tree (*Melaleuca alternifolia*), catnip (*Nepeta cataria*) and Indian borage (*Plectranthus amboinicus*) [12,13,39], have also been reported to show increased insecticidal activities with higher concentrations and longer exposure times. Moreover, susceptibility to the essential oil can vary according to the sex of the fly [40]. Sukontason et al. [40] reported that the males of *M. domestica* and *C. megacephala* are more susceptible to eucalyptol than females because they are usually smaller in size. However, the influence of sex on insecticide susceptibility was not evaluated in our study.

In terms of toxicity values, the LD_50_ and LC_50_ values of the *C. aurantium* essential oil against *S. calcitrans* at 24 h after treatment for the contact and fumigant toxicity tests were 105.88 µg/fly or 21.17% (*w*/*v*) and 13.06 mg/L air or 13.06 µg/cm^3^ air, respectively. Upon comparing the toxicity values with the essential oils of other plants against *S. calcitrans*, we found that the *C. aurantium* essential oil had relatively higher toxicity values than other plants. For instance, the Japanese pepper (*Zanthoxylum piperitum*) and bamboo-leaf prickly ash (*Zanthoxylum armatum*) essential oils showed LD_50_ and LC_50_ of 11.058 µg/fly and 0.264 µg/cm^3^, and 26.981 µg/fly and 0.347 µg/cm^3^, respectively [41]; the tea tree essential oil had LD_50_ and LC_50_ values of 3.82 and 1.06% (*w*/*v*), respectively [12], and the Indian borage essential oil had LD_50_ and LC_50_ values of 12.05 µg/fly and 1.34 mg/L air, respectively [13]. By contrast, *C. aurantium* essential oil was found to have relatively lower fumigant toxicity value (LC_50_) than the catnip (*N. cataria*) essential oil, which showed LC_50_ in the modified K&D system and fumigant jar of 7.7 and 10.7 mg/cm^3^ [39].

The insecticidal activities of some compounds found in *C. aurantium* essential oil, such as limonene, α-pinene, and β-myrcene, have been reported. Limonene has been reported to have insecticidal activity against several insect pests, including mealybugs and scale insects [42], horn flies [43], German cockroaches [44], and tomato leafminer [45]. α-Pinene and β-myrcene were found in lower relative contents in our study. The previous studies reported that α-pinene exhibited larvicidal and adulticidal effects against *Aedes aegypti* [46] and adulticidal effect against weevil (*Sitophilus zeamais*) [47]. In addition, this compound also inhibited the development of immature stages of the weevil and reduced progeny by up to 94% [47]. β-Myrcene was found to be toxic to red flour beetle (*T. castaneum*), cigarette beetle (*Lasioderma serricorne*), and book louse (*L. bostrychophila*) in the contact toxicity test [48]. 

The combinations of some phytochemical compounds can exert insecticidal activity; limonene has a synergistic effect with α-pinene and sabinene, and sabinene has additive effects with α-pinene, 1,8-cineole, 1-octen-3-ol, and linalool [49]. In this study, we did not test the insecticidal activity of pure limonene against *S. calcitrans*. However, a previous study reported the insecticidal activity of *C. aurantium* essential oil (limonene = 87.52%) and pure limonene against adults of *T. absoluta* by contact toxicity test. The results showed that the *C. aurantium* essential oil had a relatively lower toxicity value (LC_50_ = 10.65 µL/L air) than the pure limonene (LC_50_ = 37.36 µL/L air) [25]. These results suggest that limonene and the other constituents of *C. aurantium* essential oil may be responsible for its strong insecticidal activities. Interestingly, Showler et al. [43] found that a low concentration of limonene (<0.1%) might be useful for trapping the horn fly and its insect attractant properties. 

This study demonstrated that the fumigant toxicity of *C. aurantium* essential oil was relatively more effective against stable flies with a lower concentration. Essential oils are largely responsible for fumigant action and may exert toxicity by penetrating the insect body via the respiratory system [50], the cuticle, or through the digestive system [50,51]. Essential oils are also lipophilic and may affect the insect nervous system, thereby causing insect paralysis and death. The possible target mechanisms are the inhibition of acetylcholinesterase and its positive allosteric modulation of the gamma-aminobutyric acid and metabotropic octopamine receptors [52]. In addition, *C. aurantium* essential oil might also exhibit its effect on neurotransmitters. A previous study revealed that *C. aurantium* essential oil binds to both the acetylcholinesterase enzyme (AChE) and the enzyme substrate, leading to the accumulation of acetylcholine at the synapses. In turn, this causes the post-synaptic membrane to be stimulated all the time, resulting in a general loss of coordination in the neuromuscular system and eventually, death [45]. Additionally, α-pinene showed strong AChE inhibition activity against rice weevil (*Sitophilus oryzae*) [53]. 

*C. aurantium* essential oil is considered safe for application in mammals. Studies on toxicity revealed that oral treatment with *C. aurantium* essential oil at 500 mg/kg for 14 days in pregnant Wistar rats did not interfere with maternal reproductive performance, body weight gain, water intake, and food consumption and caused no teratogenic effect [54]. Furthermore, oral administration to albino mice with *C. aurantium* essential oil at a concentration of 2000 mg/kg did not elicit any clinical symptoms of acute toxicity or mortality in any of the mice. There were also no changes in food intake, behavior, or body weight during the monitoring period (14 days) [55]. 

Although essential oils have undesirable side effects as natural products, they also possess low toxicity against humans and animals and are rapidly biodegradable; thus, they are increasingly being used to replace synthetic chemicals as green pesticides [56]. From this study, it was found that *C. aurantium* oil has insecticidal properties that have toxic effects on stable fly and could be used as an alternative bioinsecticide. 

## 4. Materials and Methods

### 4.1. Ethical Statement

The study protocol was approved by the Faculty of Veterinary Science, Mahidol University Animal Care and Use Committee (Ref. MUVS-2020-12-63).

### 4.2. Insect

Wild-caught specimens of stable flies were used in this study, which followed the WHO susceptibility test guidelines [28]. To reduce the factors related to the physiological status of specimens, only nonblood-fed specimens were used for testing. The specimens were collected from a horse farm in Nakhon Pathom Province, Central Thailand (13°45′43.4″ N 100°08′15.7″ E), between March and May 2021. This farm did not use insecticides. The Nzi traps [57] were placed at the collection site from 16:00 to 18:00. The collected flies were stored in plastic cups and then transported within Styrofoam boxes containing ice packs to the Pharmacology Laboratory of the Faculty of Veterinary Science, Mahidol University. Both male and female stable flies were used for insecticide testing. In particular, the nonblood-fed specimens with undamaged physical characteristics (i.e., antenna, wing, and leg) were selected from all collected specimens under a stereomicroscope (SMZ745, Nikon, Tokyo, Japan) without anesthesia. In the laboratory, these specimens were maintained at 27–29 °C and 70–80% relative humidity until they were used for testing (within 1–2 h).

### 4.3. Essential Oil Extraction and Analysis

Insecticide-free *Citrus aurantium* var. *aurantium* was obtained from homegrown plants in Chainat Province, Central Thailand (14°58′18.0″N 100°16.′02.0″E). The plant was identified and deposited at the Department of Pharmaceutical Botany, Faculty of Pharmacy, Mahidol University (PBM No. 005495-6). Essential oil was extracted from 8 kg of fresh peels of unripe fruits using the steam distillation method, which was conducted for 6 h. The extracted essential oil was stored in amber glass bottles at 4 °C until use. Essential oil yield was calculated in % (*v*/*w*) based on the weight of the fresh peel material.

The physical properties of the essential oil were determined as follows: the color was evaluated by visual inspection, pH was measured with pH indicator strips (Merck, Darmstadt, Germany), density was measured with a density meter (DA-100M, Tokyo, Japan), and the refractive index was calculated with the use of a refractometer (RX-5000CX, Atago, Tokyo, Japan).

The chemical constituents in the essential oil were determined by gas chromatography-mass spectrometry (GC-MS) (Model 7890A-MS5975C, Agilent Technologies, Santa Clara, CA, USA) equipped with a DB-5HT capillary column (length: 30 m, inner diameter: 0.25 mm, and film thickness: 0.1 µm; Agilent Technologies, USA). The essential oil sample was injected in the split mode, with a 1:10 split ratio. Helium was used as the carrier gas, at a flow rate of 1 mL min^−1^. The temperature of the injection port was set at 250 °C, and the column temperature program was as follows: 50 °C for 2 min, followed by an increase to 250 °C at a rate of 10 °C min^−1^, after which the temperature was maintained at 250 °C for 5 min. The mass spectrometry conditions consisted of the following: ion source temperature of 230 °C, ionization energy of 70 eV, and mass scan range of 350–550 amu. The constituents were identified by comparison of their mass spectra with data in Wiley 7N edition (Mass Spectra library). We calculated the concentration of the main constituents by comparing the peak area of sample with the peak area of standard.

### 4.4. Contact Toxicity Test

The contact toxicity of essential oil from *C. aurantium* against stable flies was tested by topical application, according to the procedure of Leesombun et al. [13]. Preliminary studies were conducted to determine the appropriate test range concentrations causing 10–90% mortality. Essential oil was diluted in acetone to obtain five concentrations: 42, 84, 210, 420, and 840 µg/µL. A total of 210 stable flies of mixed sexes were anesthetized at −20 °C for 30–45 s. Then, using a micropipette, 0.5 µL of each concentration, ranging from 21 to 420 µg/fly, was applied directly on the thorax. Acetone [39] and cypermethrin 1% (*w*/*v*) were used as negative and positive controls, respectively, and were applied in the same volumes. Each treatment was performed with 10 flies in three replications. The treated flies were placed in a sterile, transparent plastic cup (11 cm diameter, 8.5 cm height) covered with mesh fabric secured with rubber bands. As a source of energy, a 10% (*v*/*v*) honey solution on cotton wool was provided at the top of the mesh fabric. Flies were allowed to recover at temperatures ranging from 27–29 °C and 70–80% relative humidity. The mortality rates were recorded at 1, 2, 4, 6, 12, and 24 h after treatment. The flies were considered dead when they did not respond after mechanical stimulation with a paintbrush.

### 4.5. Fumigant Toxicity Test 

The fumigant toxicity of essential oil from *C. aurantium* against stable flies was assessed according to the protocols described by Leesombun et al. [13]. This test was conducted in a 1 L sterile, transparent plastic box with a lid. Preliminary studies were conducted to determine the appropriate test range of concentrations causing 10–90% mortality. Different amounts of essential oil (0.84, 4.20, 8.40, 16.80, and 25.20 mg) dissolved in 100 µL of acetone were separately pipetted onto 55 mm diameter Whatman No. 1 filter papers (GE Healthcare, Buckinghamshire, UK), which were then placed onto the bottom of a glass Petri dish (diameter 55 mm). The solvent on each filter paper was allowed to evaporate for 2–3 min, after which the Petri dish was covered with mesh fabric secured with rubber bands to prevent contact between the filter paper and the flies. Acetone [39] and cypermethrin 1% (*w*/*v*) were used as negative and positive controls, respectively. Next, the Petri dishes were placed on the bottom of a plastic box. A 10% (*v*/*v*) honey solution on cotton wool was also placed at the bottom of each box. For the testing, a total of 210 stable flies of mixed sexes were anesthetized at −20 °C for 30–45 s and then placed in a plastic box before it was closed securely. Each treatment was performed with 10 flies in three replications. The flies were allowed to recover and were maintained at temperatures ranging from 27–29 °C with 70–80% relative humidity. We recorded the mortality rates at 1, 2, 4, 6, 12, and 24 h after treatment. The flies were considered dead when they no longer showed movement.

### 4.6. Statistical Analysis 

The toxicity tests considered by over 20% of control mortality were discharged and repeated. If control mortality was greater than 5%, the observed mortality was corrected using Abbott’s formula [58]. All variables were tested for normality and homogeneity of the variance using the Shapiro–Wilk and Levene tests, respectively. We analyzed the statistical comparisons of the mortality between treatments and controls by one-way analysis of variance (ANOVA) followed by Tukey’s HSD test in SPSS version 21.0 software (SPSS, Chicago, IL, USA). Repeated measures ANOVA and Greenhouse-Geisser correction were used to evaluate the effects of the treatments and exposure times on the mortality with SPSS version 21.0 software. The repeated factor was exposure time, whereas the response variable was insect mortality, and the main effect was treatment. A *p*-value < 0.05 was considered statically significant. Probit analysis for calculating toxicity values, including median lethal dose (LD_50_) and 90% lethal dose (LD_90_) at 24 h after treatment and median lethal concentrations (LC_50_) and 90% lethal concentration (LC_90_) at 24 h after treatment, was performed using LdP line Software (Ehab Mostafa Bakr, Dokki, Cairo, Egypt), freely downloaded at http://www.ehabsoft.com/ldpline/, accessed on 1 November 2021.

## 5. Conclusions

This study demonstrated the activity of *C. aurantium* essential oil, which contains considerable amounts of limonene, as an insecticide against the stable fly (*S. calcitrans*) based on contact toxicity and fumigant toxicity tests. Our results showed that *C. aurantium* essential oil exhibited both contact and fumigant activities against *S. calcitrans*. Therefore, this essential oil could be used as an alternative to synthetic insecticides for stable fly control. However, to compare our findings, further investigations are required to evaluate flies obtained from laboratory colonies. Additionally, the development of essential oil formulations through synergistic combinations of compounds is required to increase the effectiveness of essential oil.

## Figures and Tables

**Table 1:** Chemical composition of *Citrus aurantium* essential oil.

No.	Retention Time (min)	Compounds	Peak Area (%)	% Similarity Index
1	4.10	α-Pinene	0.56	97
2	4.78	Sabinene	0.23	97
3	5.10	β-Myrcene	1.99	97
4	5.32	Octanal	0.52	92
5	5.65	Limonene	93.79	98
6	9.71	Neryl acetate	0.13	80
7	11.78	Germacrene	0.48	99
8	24.50	Bis (2-ethylhexyl) phthalate	0.09	72
**Total**			**97.79**	

**Table 2:** Percent mortality (mean ± SD) of insecticidal activity of *Citrus aurantium* essential oil against *Stomoxys calcitrans* by contact toxicity test at different concentrations.

Concentration (µg/µL)	Mortality (%)					
	1 h	2 h	4 h	6 h	12 h	24 h
Negative control	0 ^a^	0 ^a^	0 ^a^	0 ^a^	0 ^a^	0 ^a^
Cypermethrin (1%)	100 ^d^	100 ^d^	100 ^d^	100 ^d^	100 ^d^	100 ^c^
42	0 ^a^	0 ^a^	6.7 ± 5.8 ^a^	6.7 ± 5.8 ^a^	10.0 ± 0.0 ^a^	10.0 ± 0.0 ^a^
84	0 ^a^	0 ^a^	0 ^a^	0 ^a^	6.7 ± 5.8 ^a^	20.0 ± 0.0 ^a^
210	30.0 ± 10.0 ^b^	30.0 ± 10.0 ^b^	30.0 ± 10.0 ^b^	30.0 ± 0.0 ^b^	33.3 ± 5.8 ^b^	46.7 ± 10.0 ^b^
420	63.3 ± 5.8 ^c^	66.7 ± 5.8 ^c^	66.7 ± 5.8 ^c^	73.3 ± 11.6 ^c^	76.7 ± 5.8 ^c^	80.0 ± 0.0 ^c^
840	80.0 ± 17.3 ^cd^	80.0 ± 17.3 ^cd^	83.3 ± 11.6 ^cd^	83.3 ± 11.6 ^cd^	83.3 ± 11.6 ^cd^	83.3 ± 11.6 ^c^
df	6, 14	6, 14	6, 14	6, 14	6, 14	6, 14
F	85.923	87.308	123.593	96.028	155.238	58.725
*p*	<0.001	<0.001	<0.001	<0.001	<0.001	<0.001

Statistically significant differences (*p* < 0.05) are indicated by different letters.

**Table 3:** Lethal dose (LD_50_ and LD_90_) of *Citrus aurantium* essential oil against *Stomoxys calcitrans* by contact toxicity test at 24 h after treatment.

Treatment	Contact Toxicity Test
LD_50_ [µg/fly] (95% CL)	105.88 (79.42–141.88)
LD_90_ [µg/fly] (95% CL)	499.25 (324.49–1018.42)
Slope ± SE	1.9 ± 1.73
χ^2^	1.73

**Table 4:** Percent mortality (mean ± SD) of insecticidal activity of *Citrus aurantium* essential oil against *Stomoxys calcitrans* by fumigant toxicity test at different concentrations.

Concentration (mg/L Air)	Mortality (%)					
	1 h	2 h	4 h	6 h	12 h	24 h
Negative control	0 ^a^	0 ^a^	0 ^a^	0 ^a^	0 ^a^	0 ^a^
Cypermethrin (1%)	100 ^c^	100 ^c^	100 ^c^	100 ^c^	100 ^c^	100 ^c^
0.84	0 ^a^	0 ^a^	0 ^a^	0 ^a^	3.3 ± 5.8 ^a^	3.3 ± 5.8 ^a^
4.20	0 ^a^	0 ^a^	0 ^a^	3.3 ± 5.8 ^a^	6.7 ± 5.8 ^a^	6.7 ± 5.8 ^a^
8.40	0 ^a^	6.7 ± 11.6 ^ab^	13.3 ± 11.6 ^a^	20.0 ± 20.0 ^ab^	23.3 ± 15.3 ^ab^	23.3 ± 15.3 ^ab^
16.80	20.0 ± 10.0 ^a^	30.0 ± 10.0 ^b^	43.3 ± 15.3 ^b^	43.3 ± 15.3 ^b^	43.3 ± 15.3 ^b^	53.3 ± 15.3 ^b^
25.20	60.0 ± 20.0 ^b^	80.0 ± 20.0 ^c^	83.3 ± 15.3 ^c^	90.0 ± 10.0 ^c^	90.0 ± 10.0 ^c^	90.0 ± 10.0 ^c^
df	6, 14	6, 14	6, 14	6, 14	6, 14	6, 14
F	41.000	58.789	62.833	50.116	57.579	39.786
*p*	<0.001	<0.001	<0.001	<0.001	<0.001	<0.001

Statistically significant differences (*p* < 0.05) are indicated by different letters.

**Table 5:** Lethal concentration (LC_50_ and LC_90_) of *Citrus aurantium* essential oil against *Stomoxys calcitrans* by fumigant toxicity test at 24 h after treatment.

Treatment	**Fumigant Toxicity Test**
LC_50_ [mg/L air] (95% CL)	13.06 (5.41–62.53)
LC_90_ [mg/L air] (95% CL)	43.13 (n/a)
Slope ± SE	2.47 ± 0.43
χ^2^	24.68

n/a = not available.

## Data Availability

The data presented in this study are available within the article.

## Supplementary Materials

The following supporting information can be downloaded at: https://www.mdpi.com/article/10.3390/plants11091122/s1, Figure S1: Mass spectra of compounds in *Citrus aurantium* essential oil with standard mass spectra from Wiley 7N edition (Mass Spectra library).
