# Supplementary material for: Contact and Fumigant Activities of Citrus aurantium Essential Oil against the Stable Fly Stomoxys calcitrans (Diptera: Muscidae)
Source: Plants (Basel). 2022 Apr 21;11(9):1122. doi: 10.3390/plants11091122 (PMC9102086; doi:10.3390/plants11091122)
Supplement: Supplementary file 1 [file plants-11-01122-s001.zip › Figure S1.pdf]

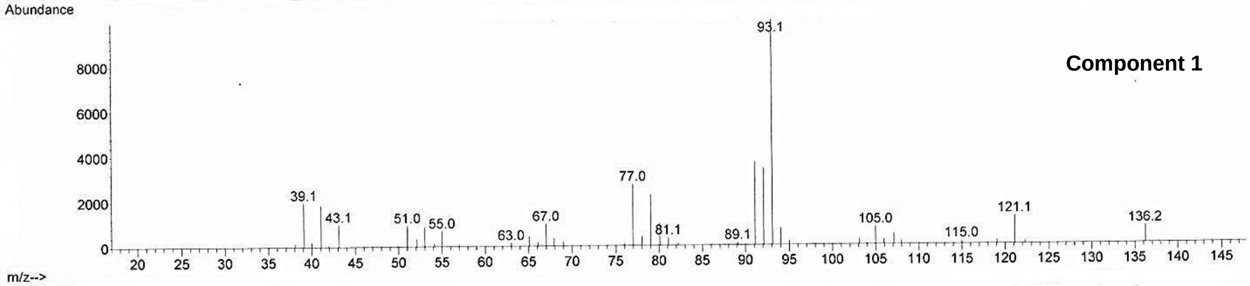
Abundance
Component 1
93.1
77.0
39.1
43.1
51.0
55.0
63.0
67.0
81.1
89.1
105.0
115.0
121.1
136.2
m/z-->

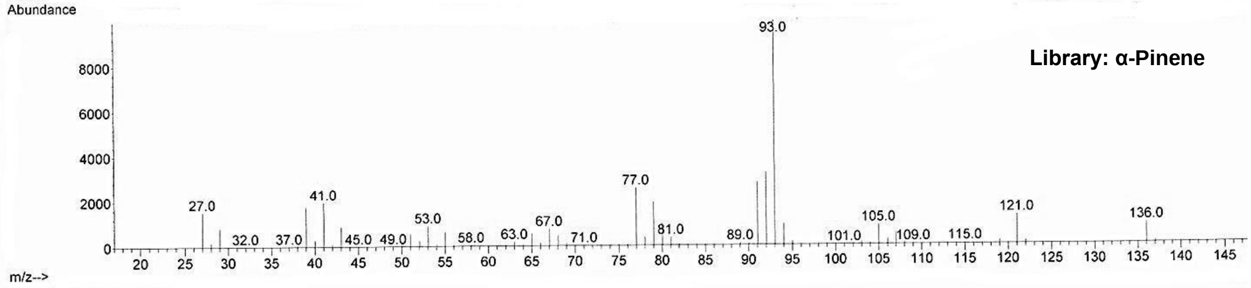
Abundance
Library: α-Pinene
93.0
77.0
41.0
27.0
32.0
37.0
45.0
49.0
53.0
58.0
63.0
67.0
71.0
81.0
89.0
101.0
105.0
109.0
115.0
121.0
136.0
m/z-->

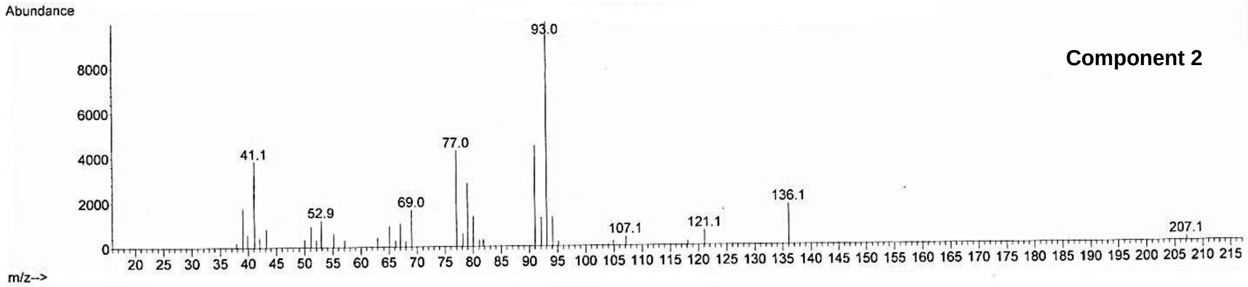
Abundance
Component 2
93.0
41.1
77.0
52.9
69.0
107.1
121.1
136.1
207.1
m/z-->

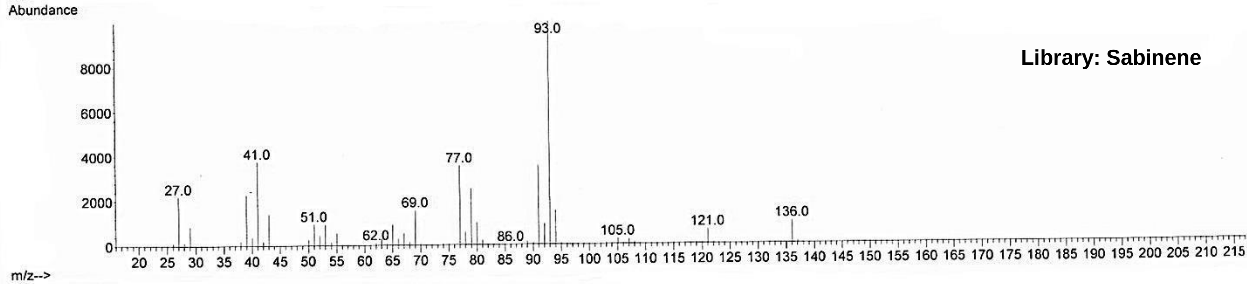
Abundance
Library: Sabinene
93.0
41.0
77.0
27.0
51.0
62.0
69.0
86.0
105.0
121.0
136.0
m/z-->

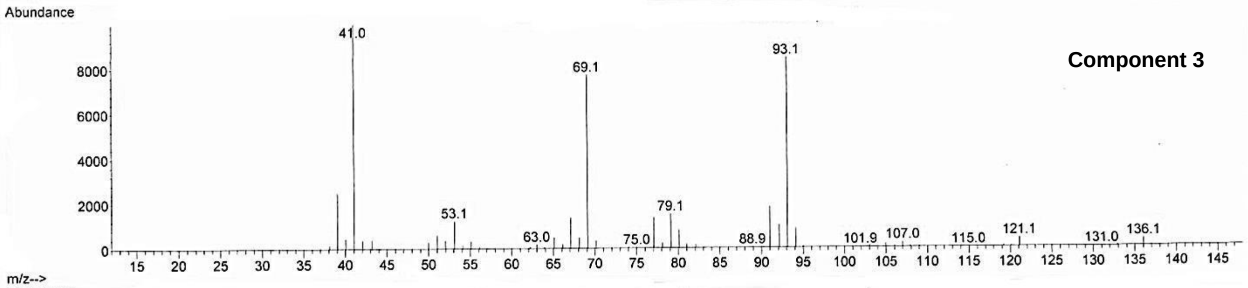
Abundance
Component 3
41.0
69.1
93.1
53.1
63.0
75.0
79.1
88.9
101.9
107.0
115.0
121.1
131.0
136.1
8000
6000
4000
2000
0
15 20 25 30 35 40 45 50 55 60 65 70 75 80 85 90 95 100 105 110 115 120 125 130 135 140 145
m/z-->

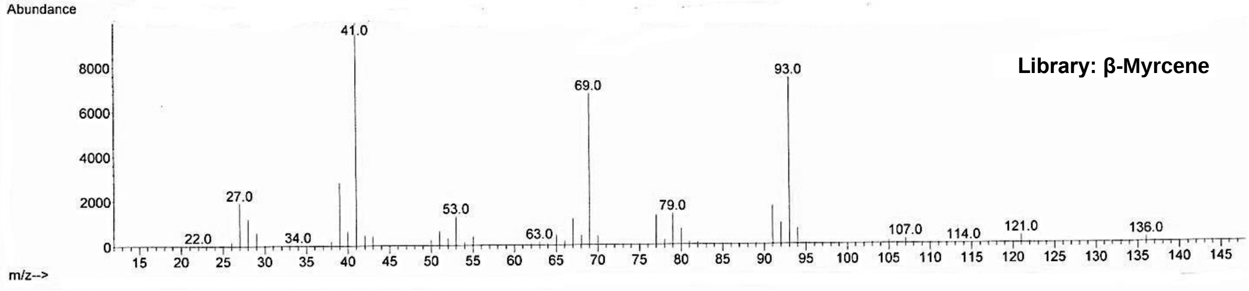
Abundance
Library: β-Myrcene
41.0
69.0
93.0
27.0
22.0
34.0
53.0
63.0
79.0
107.0
114.0
121.0
136.0
8000
6000
4000
2000
0
15 20 25 30 35 40 45 50 55 60 65 70 75 80 85 90 95 100 105 110 115 120 125 130 135 140 145
m/z-->

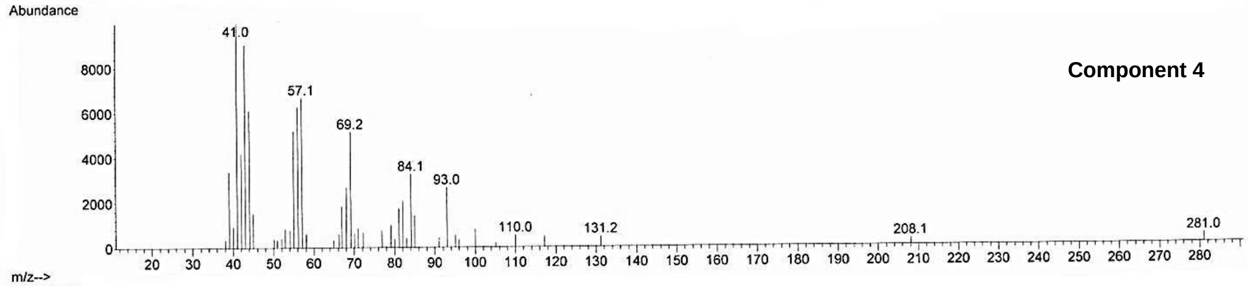
Abundance
Component 4
41.0
57.1
69.2
84.1
93.0
110.0
131.2
208.1
281.0
8000
6000
4000
2000
0
20 30 40 50 60 70 80 90 100 110 120 130 140 150 160 170 180 190 200 210 220 230 240 250 260 270 280
m/z-->

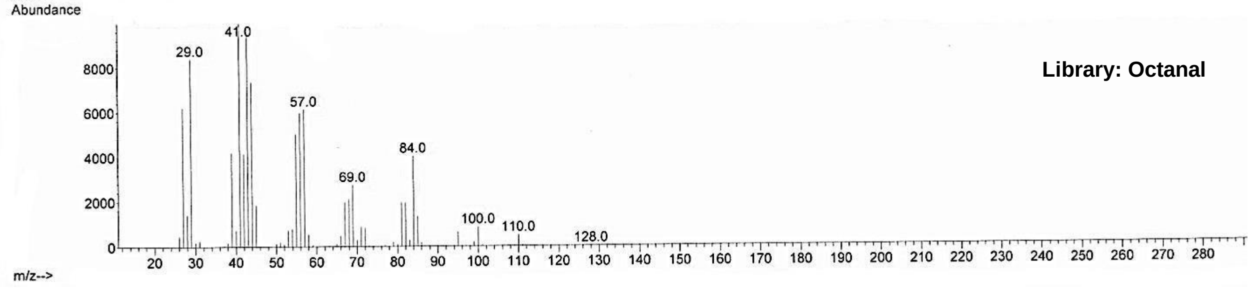
Abundance
Library: Octanal
29.0
41.0
57.0
69.0
84.0
100.0
110.0
128.0
8000
6000
4000
2000
0
20 30 40 50 60 70 80 90 100 110 120 130 140 150 160 170 180 190 200 210 220 230 240 250 260 270 280
m/z-->

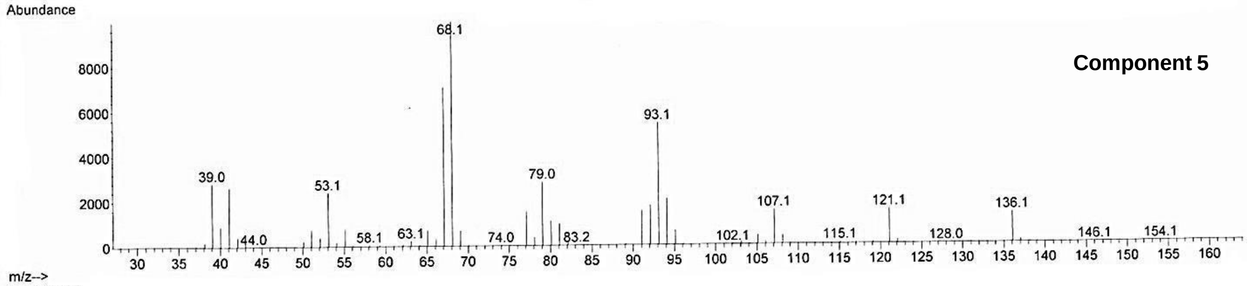
Component 5
Abundance
m/z-->
68.1
93.1
39.0
53.1
79.0
107.1
121.1
136.1
44.0
58.1
63.1
74.0
83.2
102.1
115.1
128.0
146.1
154.1

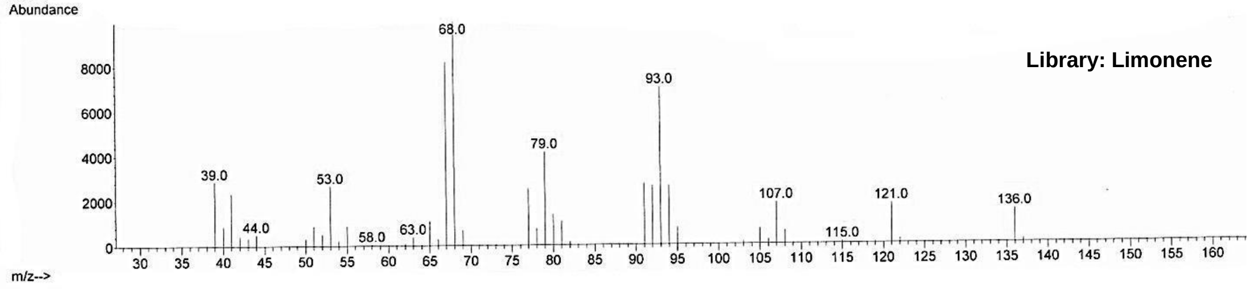
Library: Limonene
Abundance
m/z-->
68.0
93.0
79.0
39.0
53.0
107.0
121.0
136.0
44.0
58.0
63.0
115.0

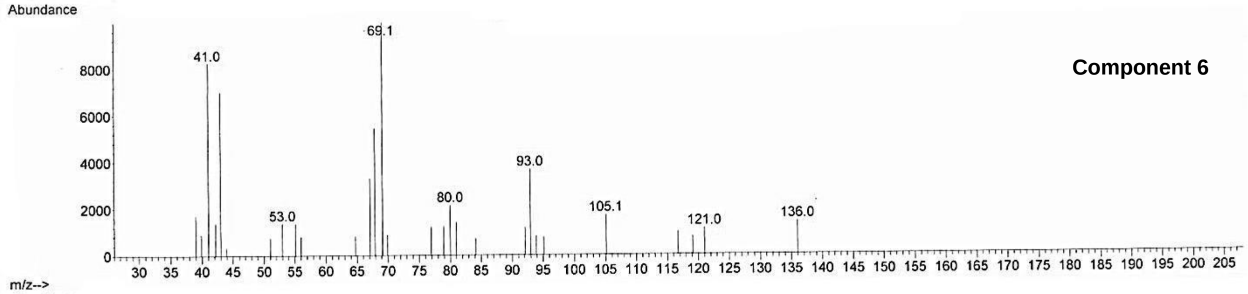
Component 6
Abundance
m/z-->
69.1
41.0
93.0
80.0
53.0
105.1
121.0
136.0

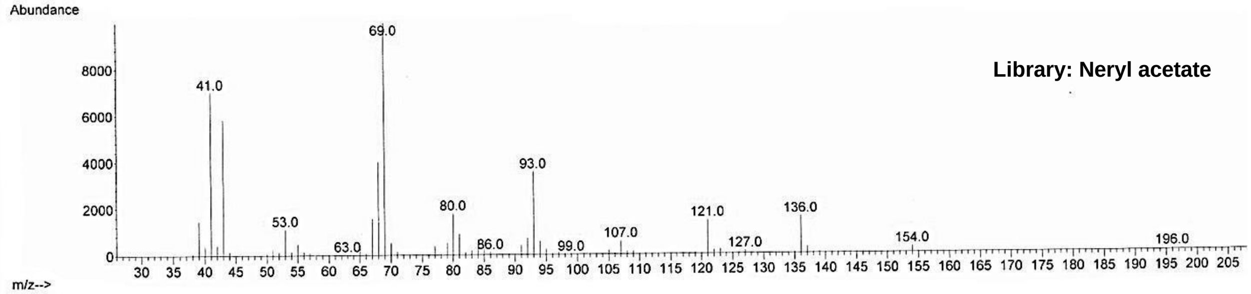
Library: Neryl acetate
Abundance
m/z-->
69.0
41.0
93.0
80.0
53.0
121.0
136.0
63.0
86.0
99.0
107.0
127.0
154.0
196.0

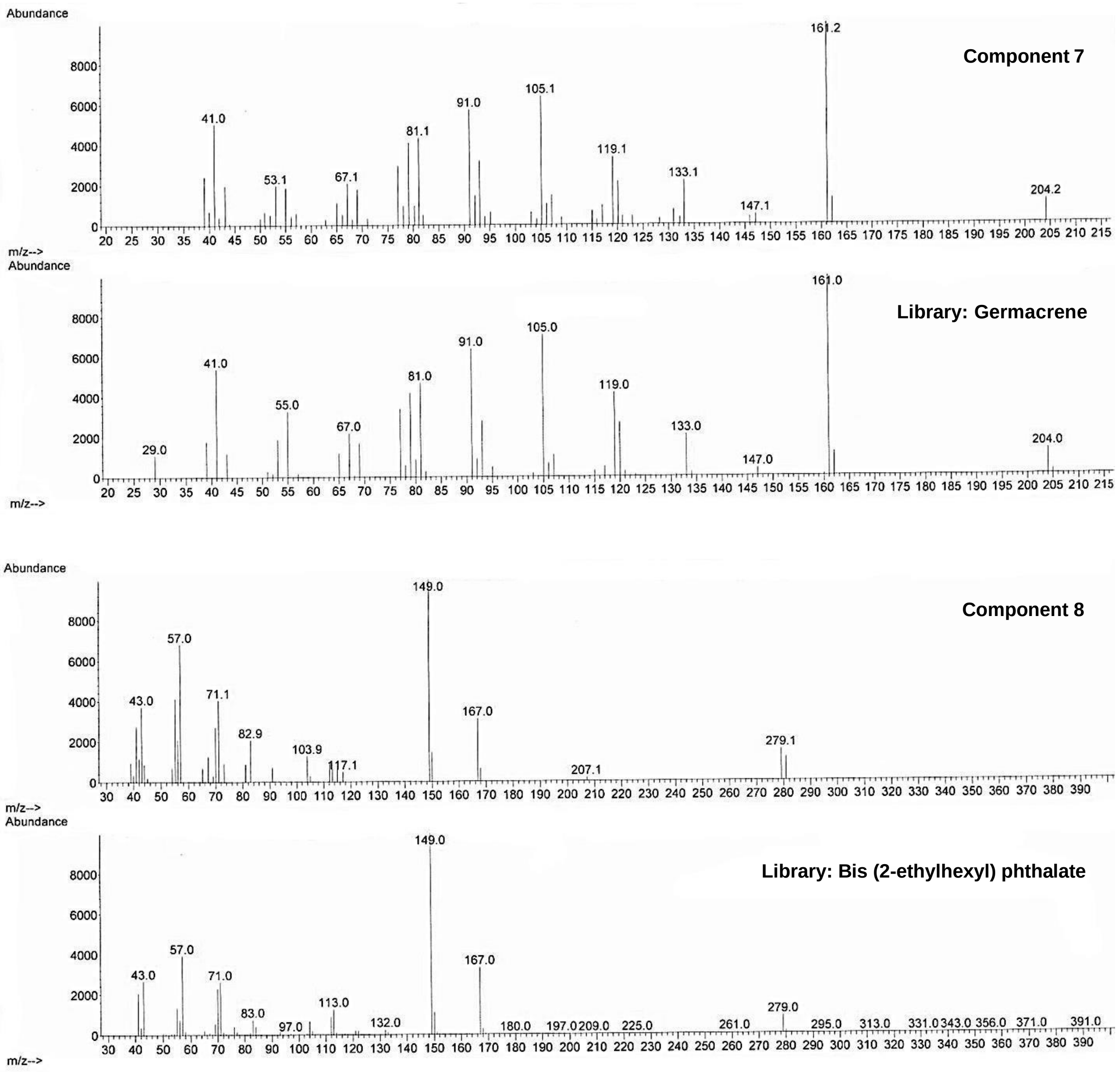

Figure S1. Mass spectra of compounds in *Citrus aurantium* essential oil with standard mass spectra from Wiley 7N edition (Mass Spectra library)
